# Concurrent Pacemaker Lead Perforation and Subacute Coronary Stent Thrombosis: A Case Report

**DOI:** 10.7759/cureus.40476

**Published:** 2023-06-15

**Authors:** Omar Shaikh, Hafsa Shaikh, Berkay Karahacioglu

**Affiliations:** 1 Cardiology, University Hospitals Dorset NHS Foundation Trust, Bournemouth, GBR; 2 Medicine, Barts Health NHS Trust, London, GBR; 3 Medicine, University College London Hospitals NHS Foundation Trust, London, GBR

**Keywords:** pacemaker complication, acute chest pain, post pci, pacemaker lead perforation, subacute stent thrombosis

## Abstract

Stent thrombosis and lead perforation are important differentials for patients presenting with chest pain following recent coronary stent insertion and pacemaker insertion. In this report, we describe an unusual case of a 78-year-old male who presented with sharp chest pain one week after admission for posterior ST-elevation myocardial infarction (STEMI) and subsequent Mobitz type II block, for which he received primary percutaneous coronary intervention (PPCI) to the left circumflex artery (LCx) and dual chamber permanent pacemaker (PPM) insertion. Computed tomography (CT) chest and CT coronary angiogram (CTCA), respectively, showed he had concurrent lead perforation and stent thrombosis. On balance, the cause of chest pain was likely lead perforation. This diagnosis was reached by having a high index of suspicion for both of these important post-procedure complications and investigating appropriately.

## Introduction

Coronary artery stent thrombosis has an incidence of 0.5-2% [1] and a mortality as high as 45% [2]. Risk factors can be divided into patient-specific (diabetes, smoking, chronic kidney disease, advanced age), coronary lesion-related (long lesion, chronic total occlusion), and procedure-related factors (multiple stents, stent under expansion).

The rates of complications relating to cardiac device therapy are variable. A 2014 study showed an overall incidence of <1% for serious complications, including lead perforation, pneumothorax, and infection [3]. Lead perforation has an incidence of <1% and, in rare cases, can be associated with death.

We present a case of concurrent stent thrombosis and lead perforation one week after stent and pacemaker insertion and address several clinical clues that may point to these diagnoses.

This article was previously posted to the Authorea preprint server on 15 March, 2023.

## Case presentation

**Figure 1:**
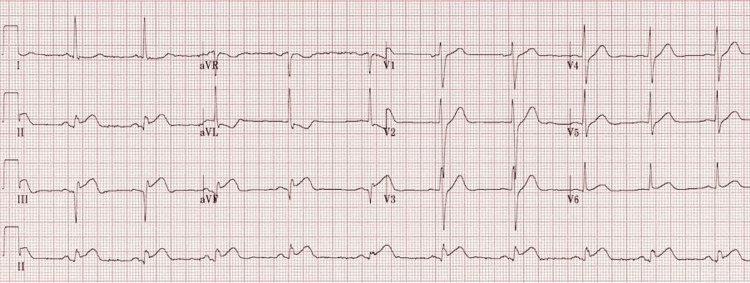
Initial ECG one week prior to this presentation showing inferior STEMI changes. STEMI: ST-elevation myocardial infarction.

**Figure 2:**
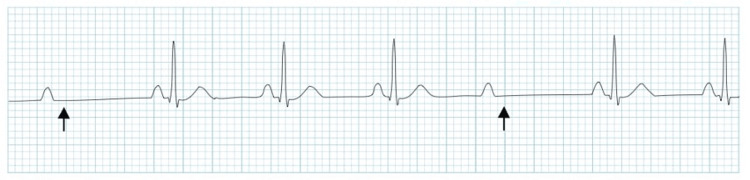
Rhythm strip showing Mobitz type II AV block. AV: atrioventricular.

A 78-year-old male with a medical history of hypertension and diabetes mellitus presented to the hospital via the primary percutaneous coronary intervention (PPCI) service with sudden onset chest pain and nausea one week after discharge from the hospital. Vital signs were within normal ranges with a respiratory rate of 20 breaths per minute, blood pressure of 136/78 mmHg, and pulse rate of 82 beats per minute. During his previous inpatient stay, he was treated for an inferior ST-elevation myocardial infarction (STEMI) with PPCI to the left circumflex artery. Post procedure, he was noted to have intermittent symptomatic Mobitz type II block without hemodynamic instability and underwent an urgent dual chamber pacemaker insertion (ECGs shown in Figures 1, 2). He was discharged on dual antiplatelet therapy and was compliant with all medications.

On arrival at the hospital, he was chest pain free. He described non-pleuritic sharp central chest pain associated with nausea and dizziness. Importantly, he reported that this chest pain felt different to the pain he had prior to his last admission with an MI.

**Figure 3:**
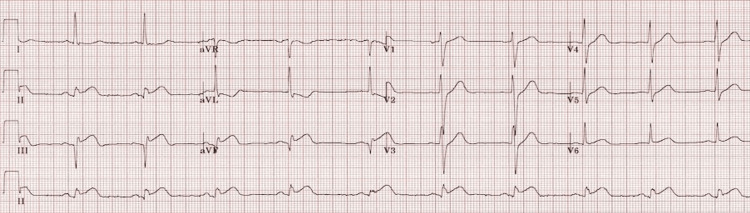
ECG on this admission again showing inferior STEMI changes, which are grossly unchanged from the previous ECG (Figure 2). STEMI: ST-elevation myocardial infarction.

An ECG showed inferior ST-segment elevation and ST depression in lead augmented vector left (aVL), with no new ischemic changes (Figure 3). Serial troponins were 1367 ng/L, 1508 ng/L, and 885 ng/L in 12-hour intervals. His initial troponin one week ago was over 20,000 ng/L.

**Figure 4:**
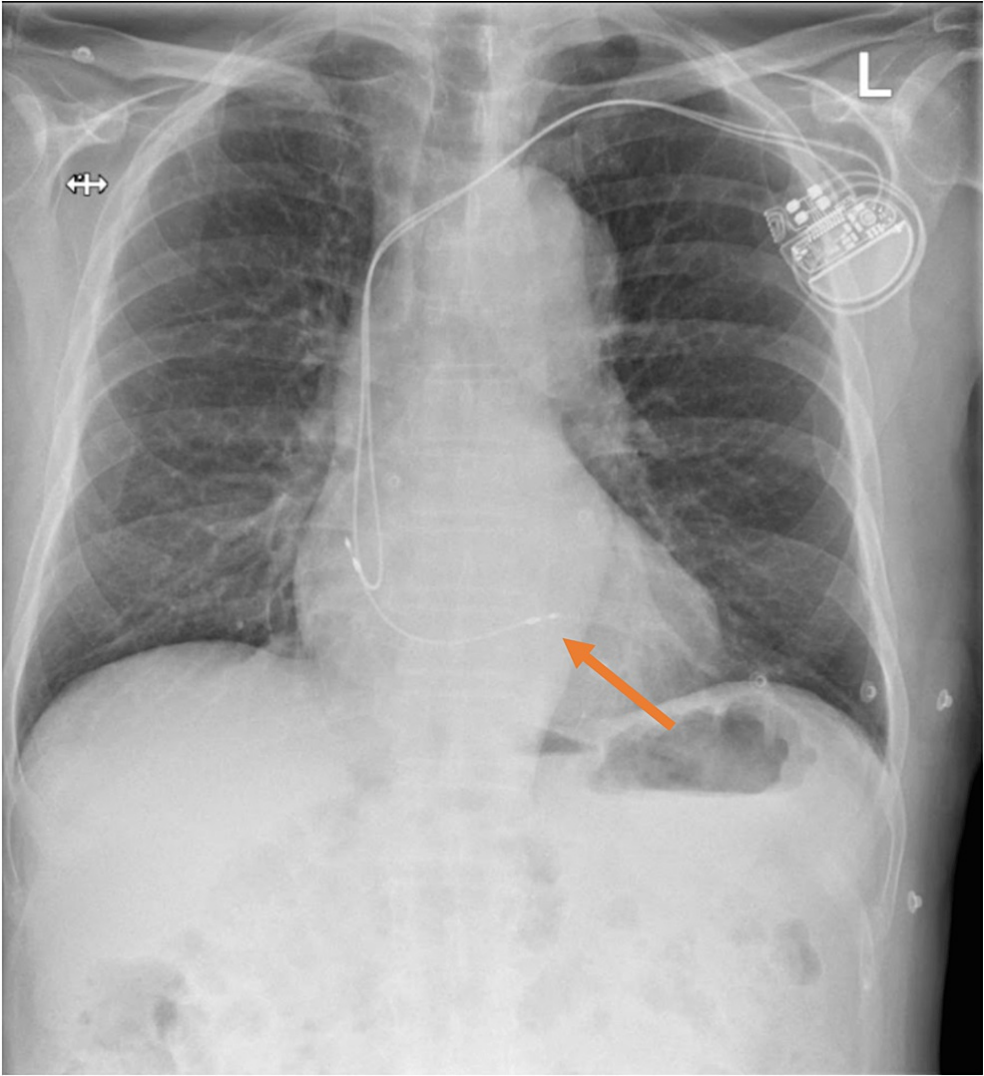
Chest radiograph showing the position of the RV pacing lead post PPM insertion. PPM: permanent pacemaker; RV: right ventricular.

**Figure 5:**
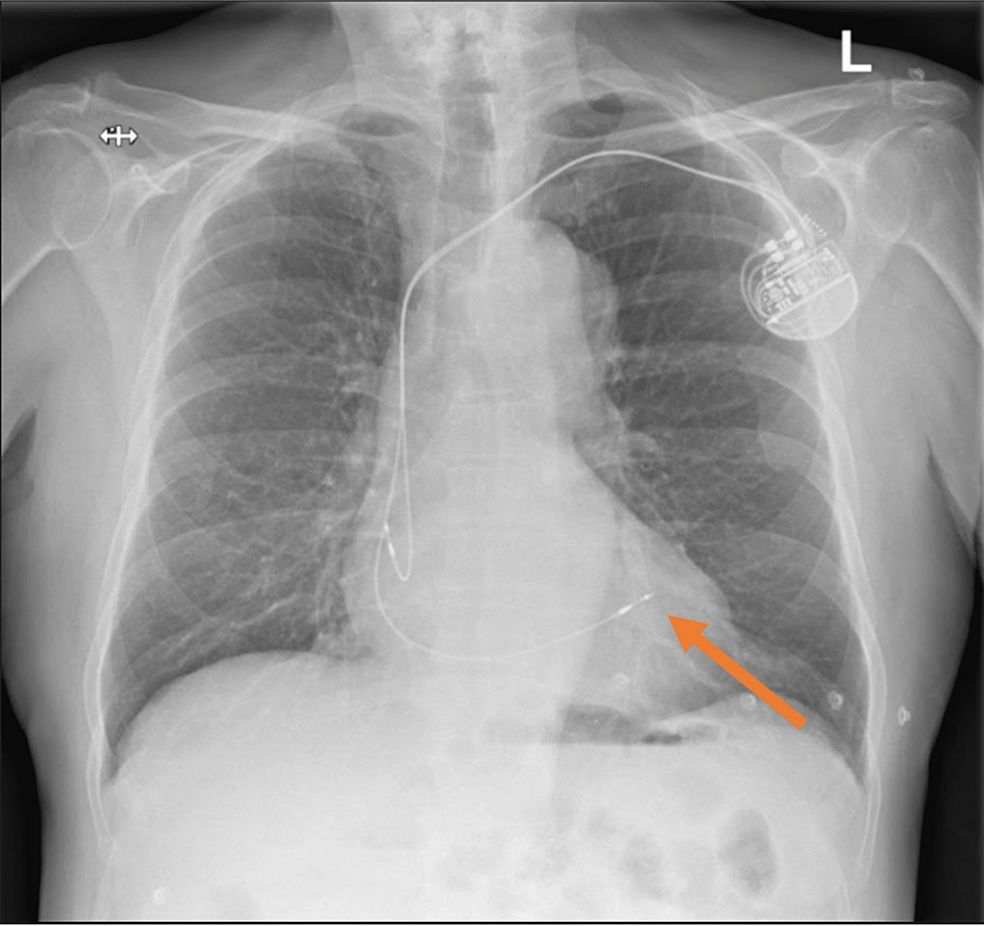
Chest radiograph on this admission. No evidence of pleural effusion/pneumothorax. The tip of the RV lead appears to be in a different position as compared to Figure 4. RV: right ventricular.

Echocardiography showed an akinetic inferolateral wall, moderately impaired left ventricular function and no evidence of pericardial effusion, much the same as his echocardiogram prior to discharge. A chest radiograph showed a subtle difference in RV lead position when compared to post-implant appearances (Figures 4, 5).

A pacemaker check showed an increased threshold (2.2) volts in bipolar mode, compared to a post-implant threshold of <1 volts. Interestingly, the lead was unable to pace in unipolar mode and would only pace the right ventricular (RV) apex in bipolar mode. This was suggestive of RV lead dysfunction and, therefore, lead perforation.

**Figure 6:**
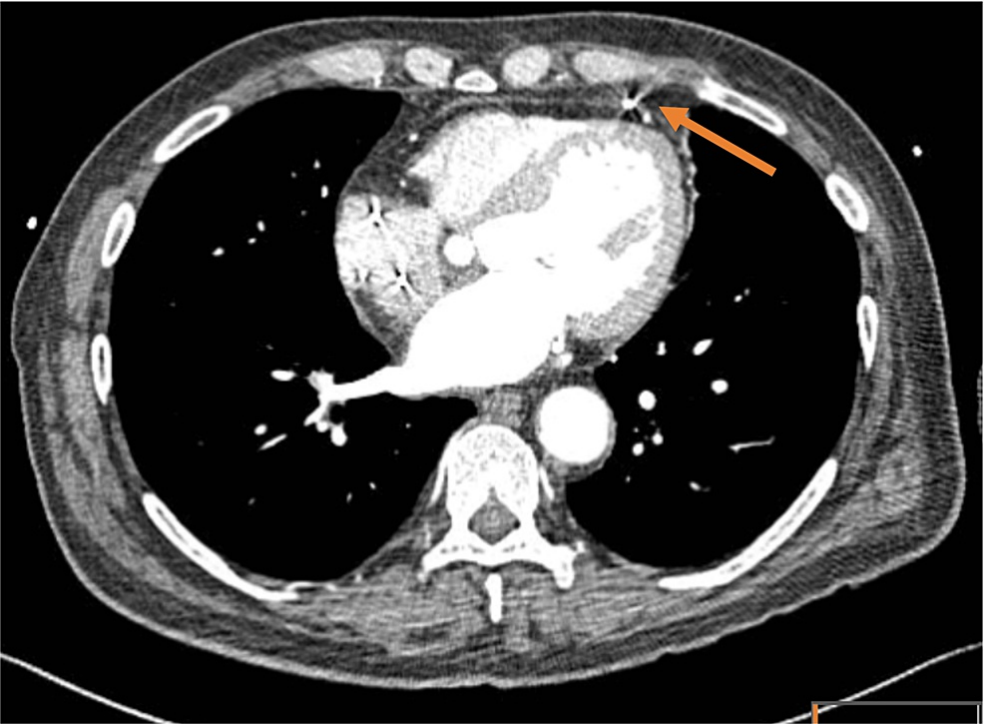
Axial slice of CT chest demonstrating RV lead perforation. RV lead is seen outside of the right ventricle abutting the pericardium. CT: computed tomography; RV: right ventricular.

A computed tomography (CT) chest and a CT coronary angiogram (CTCA) were requested to evaluate both the lead position and the status of the recently implanted coronary stent, respectively. This showed RV lead perforation and minimal pericardial effusion, with the lead protruding 10 mm to abut the pericardium (Figure 6). Surprisingly, a CTCA also showed distal occlusion of the coronary stent in the left circumflex artery.

**Figure 7:**
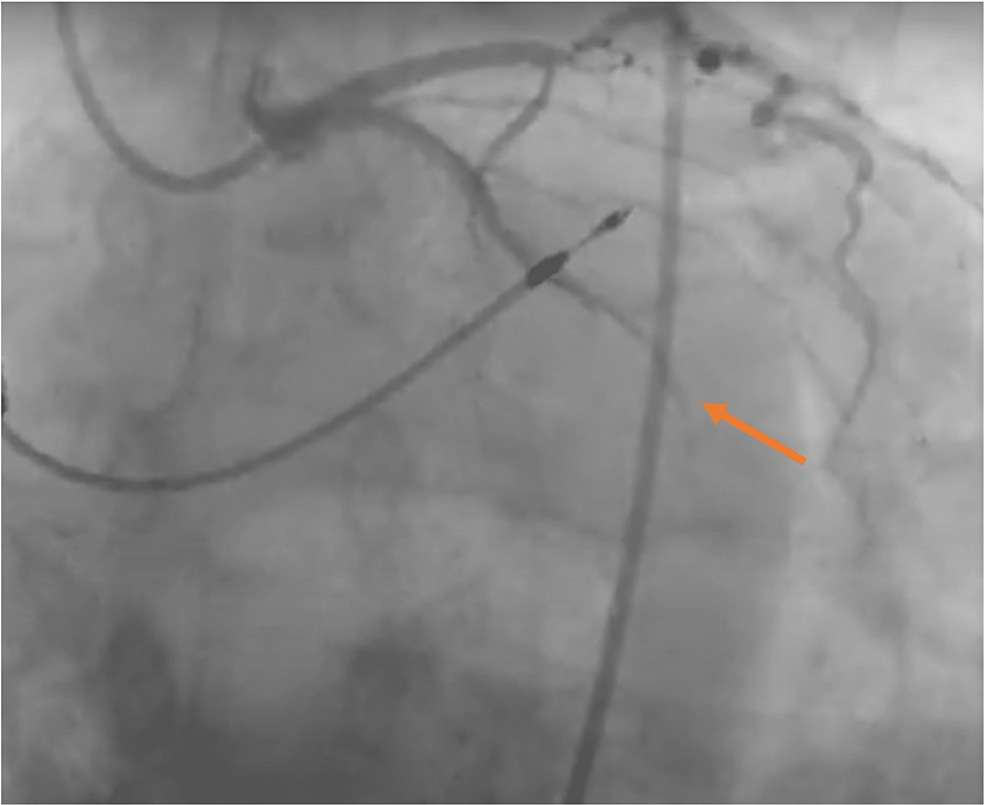
Distal occlusion of the left circumflex artery noted at CA. CA: coronary angiogram.

Following a multi-disciplinary team discussion, this patient was taken to the catheter lab to remove the perforated RV lead; a new lead was secured to the RV septum, and post procedure, pacemaker checks showed stable threshold (0.3 V) and impedance values (973 ohms). The next day, the patient underwent coronary angiography for evaluation of the distal occlusion of the stent in the left circumflex artery (Figure 7).

It proved extremely challenging to pass the guidewire beyond the middle segment of the stent. Four different angioplasty wires were used to attempt to cross the lesion without success, suggesting this was more likely subacute stent thrombosis as opposed to an acute thrombosis.

This patient was monitored for 24 hours post procedure; his echocardiogram, chest radiograph, and pacemaker check findings were satisfactory, and he was chest pain free on discharge.

## Discussion

Cardiac wall perforation secondary to pacing leads is a rare but serious and potentially fatal complication. Perforations can lead to hemopericardium, cardiac tamponade, hemothorax, coronary vessel rupture, and death [4]. The incidence of lead perforation is between 0.1 and 3%; however, clinical presentation and course range from asymptomatic to severe hemodynamic instability and death [5,6]. The electrical disturbances in the leads should be one of the first clues for the diagnosis of lead perforation [7]. In this case, a rise in the lead threshold was significant. Furthermore, the finding of failure of the pacing lead to capture in unipolar mode, but not bipolar mode, suggested that the distal part of the lead was not in contact with the apex and hence had perforated the right ventricle. Thus, when implanting septal leads during right ventricular lead placement, the operator should ensure the lead is not inadvertently placed in the RV-free wall instead. 

An echocardiogram is a useful diagnostic tool as pericardial effusion may be present, and similarly, a chest radiograph may reveal pleural effusion or show a grossly displaced lead. CT chest is regarded as the gold standard to determine the lead positions and is necessary to exclude the diagnosis of lead perforation [8].

Stent thrombosis is rare but often fatal [9]. Failure to cross the occluded lesion suggested to the operator that the stent had occluded at least a few days before the patient presented to the hospital and thus was not responsible for this presentation. In this case, it is likely that the cause of this patient’s chest pain was due to lead perforation as opposed to acute stent thrombosis for several reasons: the nature of the chest pain, no new ischemic ECG changes, failure to cross an occluded lesion, and lack of very significant troponin rise as compared to baseline troponin.

## Conclusions

Stent thrombosis may present without an ongoing troponin rise if the territory affected is already scarred and akinetic, and the threshold for CTCA should be low if there is clinical suspicion of stent thrombosis. There are several important investigations to aid diagnosis of lead perforation including chest radiographs, echocardiography, device interrogation, and CT chest.

Lead perforation and stent thrombosis are two critical diagnoses that should not be overlooked when assessing a patient with new chest pain and a history of previous stent and pacemaker insertion, and it is possible, although rare, for a patient to present with either one or both complications.
